# Assessment of Antimicrobial Use for Companion Animals in South Korea: Developing Defined Daily Doses and Investigating Veterinarians’ Perception of AMR

**DOI:** 10.3390/ani15020260

**Published:** 2025-01-17

**Authors:** Sun-Min Kim, Heyong-Seok Kim, Jong-Won Kim, Kyung-Duk Min

**Affiliations:** 1 Laboratory of Veterinary Epidemiology, College of Veterinary Medicine, Chungbuk National University, Cheongju 28644, Republic of Korea; minimins98@chungbuk.ac.kr; 2 Petobio Inc., Hanam 12982, Republic of Korea; jwkim@petobio.com

**Keywords:** one health, antimicrobial use, defined daily dose for animal, perception, veterinarians

## Abstract

There are global concerns regarding the transmission of antimicrobial-resistant pathogens from animals to humans. Especially, companion animals are increasingly recognized as a potential source due to their close interactions with people, despite a limited number of reported cases. Although, social demands regarding comprehensive surveillance for antimicrobial resistance (AMR) among companion animals are highlighted, there is a lack of a relevant system in South Korea. In this regard, we conducted preliminary investigation on antimicrobial use (AMU) among small animal clinics, along with veterinary practitioner’s knowledge and attitude regarding this issue in South Korea. We collected data on 684,153 antimicrobial prescription visits for canine and feline patients from 2019 to 2022 at 100 veterinary facilities in South Korea, using electronic medical records. To evaluate antimicrobial use (AMU) and facilitate comparisons across institutions and time periods, we developed the Defined Daily Dose for Animals (DDDA) and the Defined Animal Daily Dosages per 1000 Animal-Days (DAPD). In addition, we conducted an online survey of 362 veterinary practitioners, which included questions on their perceptions, attitudes, and practices regarding antimicrobial prescriptions. Simple frequency analyses were performed to examine temporal trends, regional differences and variations by facility size in AMU, and to summarize survey responses. Descriptive analysis using data from 100 veterinary clinics revealed a rising trend in AMU between 2019 and 2022, with higher usage observed in larger clinics and non-capital regions. DDDA values for dogs were generally higher than for cats. Survey results highlighted that, while veterinarians exhibited high awareness of AMR, prescribing practices were significantly influenced by clinical judgments and owner demands, often deviating from established guidelines. The adoption of an electronic veterinary prescription management system (e-Vet) was proposed to enhance antimicrobial stewardship. However, concerns regarding the system’s efficiency and administrative burden were prominent. To our best knowledge, this study provided DDDA for companion animals for the first time in South Korea. Although the indicator should be improved with more comprehensive data and expert opinion, our study showed that it enables reasonable situation analysis regarding AMU in companion animals. The identified factors that affect veterinarians’ prescription practices can also be used to design an effective strategy for promoting appropriate antimicrobial usage.

## Simple Summary

Animal-to-human transmission of antimicrobial-resistant pathogens is a public health issue of global concern. In particular, companion animals that have close contact with humans are a potential source, but systematic surveillance of companion animal antimicrobial use is lacking in Korea. This study evaluated the trend in antimicrobial use by category using electronic medical record data from 100 veterinary hospitals from 2019 to 2022 and developed a Defined Daily Dose for Animals for the first time in South Korea. In addition, a survey of 362 veterinary practitioners was conducted to investigate their practice regarding antimicrobial prescriptions, including awareness and behavior. The results showed an increasing trend in antimicrobial use, with higher usage observed in larger clinics and non-capital regions. Veterinarians’ awareness of antimicrobial resistance was high, but prescribing was influenced by owner demand and clinical judgment. By systematically evaluating antimicrobial use in companion animals, this study proposes a structured management framework and establishes a foundation for developing policies that support improvements in prescribing practices.

## 1. Introduction

Antimicrobial resistance (AMR) has emerged as a critical global public health issue [1]. Findings from numerous studies indicate that antimicrobial use in animals can serve as a vector for the spread of AMR [2,3,4], which emphasize appropriate antimicrobial stewardship in animals. Especially, companion animals are increasingly recognized as a potential source due to their close interactions with people [5], but only a limited number of countries have a surveillance and control program for AMR among companion animals [6].

The surveillance and control of antimicrobial use in companion animals in Korea is also insufficient. The Animal and Plant Quarantine Agency (APQA) monitors the sale of veterinary antimicrobials in Korea; however, this surveillance is primarily focused on food-producing animals [7]. Given that most antimicrobials used for the treatment of companion animals are repurposed from human medications, the current monitoring system is unlikely to fully capture every aspect of antimicrobial use in companion animals. To improve the existing system for effective management and regulation of antimicrobial use in companion animals, data comparability across variables, such as year, veterinary clinic size, and region, is crucial [8]. However, the current weight (grams)-based Korean monitoring system limits comparative data analysis. Considering that each antimicrobial has its own recommended dosage, measuring antimicrobial consumption solely by total grams makes it difficult to represent overall usage in a single, standardized measure. To overcome these limitations, the European Union introduced the Defined Daily Dose for Animals (DDDA) through the European Medicines Agency in 2015 and implemented the policy in 2016. DDDA is a concept adapted from human medicine and used for standardizing antimicrobial use based on the dose per kilogram of animal weight [9,10]. Canada, Denmark, and the Netherlands have tailored this unit for monitoring antimicrobial use in their countries [11,12,13,14].

Understanding the factors that influence veterinarians’ antimicrobial prescription patterns is essential for adopting a strategic approach to prevention of AMR. This is because veterinarians consider a variety of factors, including disease characteristics, animal age, owner expectations, and their own professional experience, when prescribing antimicrobials [15,16,17,18]. Thus, adequate support must be provided to improve the current prescribing practices to ensure that veterinarians prescribe appropriate antimicrobials and minimize the risk of AMR. However, the limited amount of research in Korea on veterinary antimicrobial prescribing patterns and their determining factors presents challenges in establishing targeted interventions.

The aim of this study was to provide a framework and evidence for the development of systematic strategies for the prevention of AMR in companion animals in Korea. Specifically, we aimed to: (1) establish the Defined Daily Dose for Animals (DDDA) in veterinary medicine, (2) assess antimicrobial usage trends in Korea from 2019 to 2022, and (3) identify the predictors of the antimicrobial prescribing practices of veterinarians in Korea.

## 2. Materials and Methods

### 2.1. Study Design and Data Collection

This study aimed to investigate antimicrobial use (AMU) and awareness in South Korean veterinary practices from 2019 to 2022 using two complementary methods: analysis of electronic medical records (EMRs) and a survey of veterinarians. The study included both veterinary and medical antimicrobials used in animal treatments, focusing on oral and injectable forms. EMRs of 100 veterinary facilities were obtained from an EMR provider (woorien^®^, Gyeonggi, Republic of Korea), which covers more than 2000 veterinary facilities in South Korea. These 100 facilities were randomly selected from among those covered by the provider. The data were provided in a de-identified format so that individual veterinary clinics could not be identified. In addition, the data were aggregated at the facility level. Therefore, individual information regarding animal patients was not identifiable. Data from 3,110,415 veterinary visits were examined, of which 684,153 visits (22.0%) involved antimicrobial prescriptions, totaling 804,800 g of antimicrobial agents. Veterinary facilities were categorized by size: small/medium (fewer than 5 veterinarians) and large (5 or more veterinarians). Geographic analysis was conducted based on South Korea’s administrative divisions (6 divisions, as presented in the results).

### 2.2. Antimicrobial Usage Analysis

To provide a comprehensive and standardized assessment of AMU, several complementary metrics were employed. These metrics ranged from basic measurements to more complex, standardized indicators. The total active ingredient quantity, expressed in grams, provided an overall volume of antimicrobials used. Prescription frequency, calculated as the percentage of visits involving antimicrobial prescriptions, offered insights into the prevalence of AMU. Standardized metrics included the DDDA and the Defined Animal Daily Dosages per 1000 Animal-Days (DAPD) [19,20]. The DDDA metric represents the average maintenance dose per day for an antimicrobial in the appropriate animal species, expressed as mg of active compound per kg of body weight (Equation (1)). DDDA values were calculated separately for canine and feline, using average weights of 6.30 kg for canine and 4.25 kg for feline (both were calculated from the collected EMR in this study). The DAPD estimates the proportion of animals treated daily with a particular antimicrobial agent. It is calculated as DDDA per 1000 animals per day, representing the number of animals per 1000 receiving a daily treatment with a particular antimicrobial (Equation (2)). For example, a DAPD of 10 indicates that an estimated 1% of the animal population receives a specific treatment on a given day. This metric allows for comparison of AMU across different animal populations and time periods.

Equation (1) is the Formula for Defined Daily Dosages for Animal (DDDA). Note that the numerator is multiplied by two, because we supposed BID in this study.


(1) $$DDDA=\frac{total\;administration\;weight\times average\;animal\;weight\times 2\;}{total\;number\;of\;administration\times total\;sum\;of\;animal\;weight\;}$$

Equation (2) is the Formula for Defined Animal Daily Dosages per 1000 Animal-Days (DAPD).


(2) $$DAPD=\frac{total\;amount\;(annually)}{\;(DDDA\times 365\;days\times total\;number\;of\;patients)}\times 1000$$

### 2.3. Survey on Veterinarians’ Awareness and Attitudes

An online survey was conducted in 2023 among a total of 362 veterinarians, representing approximately 2.6% of registered veterinary professionals in South Korea. The survey was designed to assess various aspects of AMU and AMR awareness among veterinarians. It comprised 30 questions covering AMR awareness, preventive practices, factors influencing prescribing decisions, views on antimicrobial monitoring systems, and awareness and usage of the e-Vet system (Supplementary Material). The e-Vet system, a Veterinary Prescription Management System for monitoring AMU in animals, was included in the survey to assess veterinarians’ recognition and utilization of this tool. Questions were developed based on a comprehensive literature review. The survey was conducted online, ensuring broad participation and secure, anonymous data collection and ethical approval was obtained from the Institutional Review Board (IRB) prior to survey administration (CBIRB-202305-HR-010).

### 2.4. Statistical Analysis

Simple frequency analysis was performed to characterize AMU patterns and survey responses. Both tables and figures were used for the presentation of temporal trends or comparisons by characteristics of the veterinary facilities. Data confidentiality was maintained through de-identification. Microsoft Excel (Microsoft 365, Version 2411) was used for data organization, and R software version 4.3.3 was used for performing frequency analysis and creating figures.

## 3. Result

### 3.1. Trends in Antimicrobial Usage

Antimicrobial usage data from 2019 to 2022 obtained from 100 selected veterinary clinics were analyzed (Table 1). A total of 684,153 antimicrobial prescription visits, with an annual average of 1710 visits per hospital, regardless of size or location, were recorded during the study period.

A total of 804,800 g of antimicrobials were used over the four-year period, with an annual average usage of 2012 g per hospital (Table 2).

Of 51 antimicrobials analyzed, the 15 most frequently prescribed active agents were identified (Table 3). These 15 active agents accounted for 92.4% of all antimicrobial prescriptions. Cefalexin (beta-lactam) was the most commonly prescribed (19.75%), followed by amoxicillin-clavulanate (beta-lactam; 18.08%), and metronidazole (nitroimidazole; 11.72%).

### 3.2. Antimicrobial Use According to Animal Species and Active Agents

The DDDA for each species (dogs and cats) was calculated using the data obtained from the 100 veterinary clinics (Table 4). Of the 51 antimicrobial agents analyzed, 48 had an established DDDA applicable to both dogs and cats. However, due to the lack of precise data on the usage of cefoxitin, neomycin, and aztreonam in dogs, the DDDA values for these antimicrobials were calculated only for cats. Most DDDA values for dogs were relatively higher than those for cats; however, the DDDA values of clarithromycin and lincomycin for cats were notably higher than those for dogs. In both species, cefotaxime had the highest DDDA, followed by piperacillin-tazobactam and fosfomycin.

### 3.3. Trends in Antimicrobial Usage According to Clinic Size and Region

**Figure 1:**
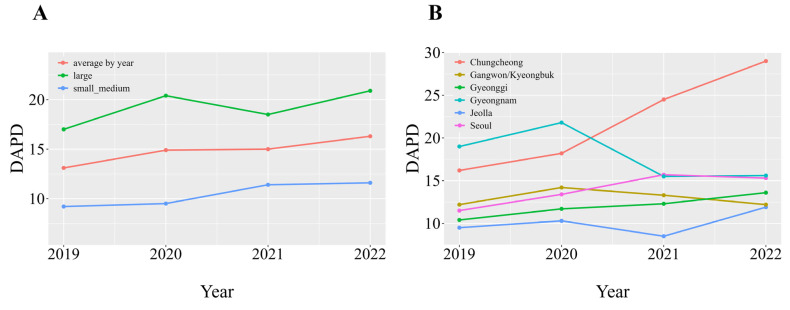
DAPD categorized according to clinic size and region. Note: (**A**) Annual average DAPD according to clinic size. The orange line indicates the overall average DAPD across all clinics. (**B**) Average DAPD according to region. DAPD, defined daily dose per population correction unit.

We analyzed antimicrobial usage patterns based on the DDDA calculated using four years (2019–2022) of electronic medical records. Antimicrobial usage showed an upward trend, from 13.1 Daily Dose per Population Correction Unit (DAPD) in 2019 to 16.3 DAPD in 2022 (Table 5, Figure 1). Regarding clinic size, the average antimicrobial usage recorded in small to medium-sized clinics was approximately 10 DAPD, whereas that in large clinics was 20 DAPD—about twice as much as that recorded in smaller clinics (Table 5). Regionally, clinics in the Chungcheong Province recorded the highest average usage (22 DAPD), which was approximately double the average recorded in Jeolla Province, which had the lowest average antimicrobial usage. Antimicrobial usage was highest in the Gyeongnam Province between 2019 and 2020 (approximately 20 DAPD); however, unlike most regions that showed a steady increase in antimicrobial use, antimicrobial usage in this region dropped sharply from 2021.

### 3.4. AMR Awareness and Prescription Practices Among Veterinarians Who Treat Companion Animals

We conducted a survey on AMR awareness and prescription practices among veterinarians who treat companion animals (Table 6). A total of 362 participants were included in the survey. Most of the respondents (48.1%) were between the ages of 30–39 years, and 71% were male. Most of the veterinarians worked in the Seoul Capital Area, with 35.9% in Gyeonggi Province (including Incheon) and 30.4% in Seoul; the remaining areas were grouped as “Other”. Most of the participants had 1–5 years of experience (37.6%), and 62.2% practiced general veterinary medicine.

### 3.5. AMR Awareness

**Figure 2:**
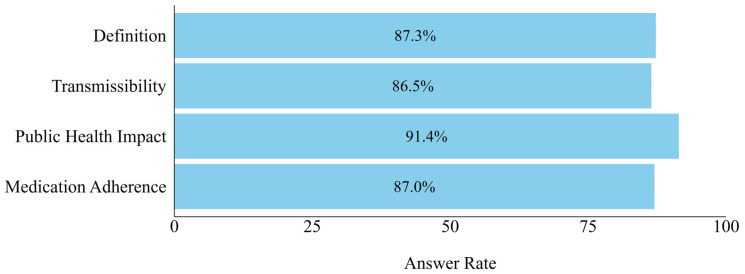
Perception of Antimicrobial Resistance and Propriate Usage. Note: “Definition” indicates proportion of correct answer regarding definition of multidrug-resistant organisms. “Transmissibility” indicates proportion of correct answer regarding transmissibility of AMR pathogen between companion animals and humans. “Public health impact” indicates proportion of participants aware of the human public health impact of AMR. “Medication compliance” indicates proportion of participants who agree that antimicrobial use should be continued even if symptoms fully improve during antimicrobial treatment.

The rate of correct responses to questions on the definition of multidrug-resistant organisms (MDROs) was 87.3%. In addition, 86.5% of the participants provided correct answers to questions on MDRO transmission. Furthermore, 87% were familiar with proper antimicrobial administration methods, and 91.2% acknowledged AMR as a public health concern (Figure 2).

### 3.6. Predictors of Antimicrobial Prescription

**Figure 3:**
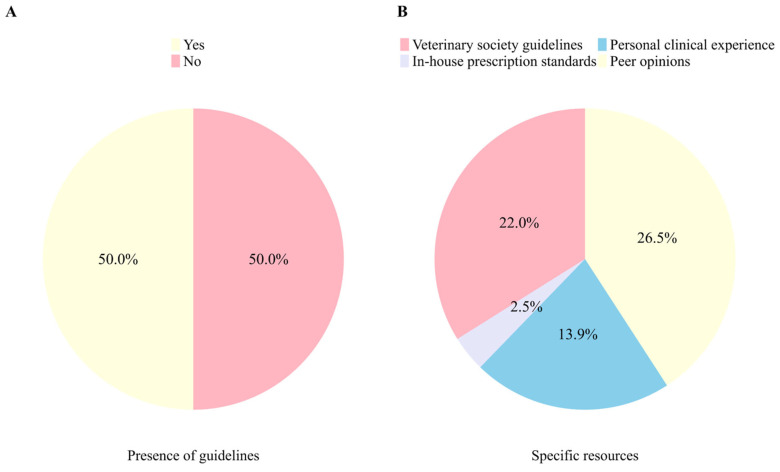
Investigating Guidelines and Resources for Antimicrobial Use in Veterinary Practices. Note: (**A**) Pie chart of responses regarding the presence of internal guidelines for antimicrobial prescription in veterinary clinics. (**B**) Pie chart of the rates of evidence utilized in antimicrobial prescribing.

We conducted a survey to identify the guidelines used by veterinarians when prescribing antimicrobials for companion animals (Figure 3). Half (50%) of the participants indicated that their facility had established guidelines for antimicrobial use (Figure 3A). Specific resources used for prescribing antimicrobials included lectures and guidelines from veterinary societies or associations (26.5%), in-house prescription standards (22%), personal clinical experience (13.9%), and peer opinions (2.5%) (Figure 3B).

**Figure 4:**
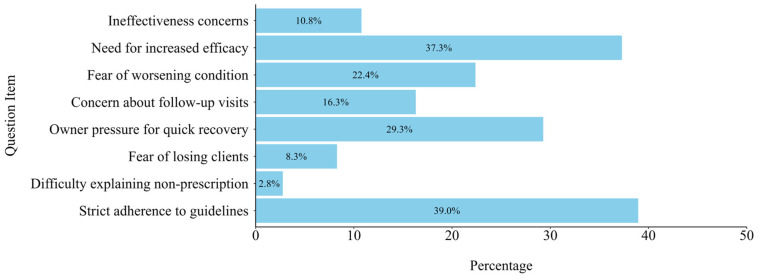
Perceived determinants for antimicrobial prescription. Note: Proportion of participants who perceived each item as determinant for antimicrobial prescription. “Ineffectiveness concerns” indicates concerns regarding the ineffectiveness of guideline-specified antimicrobials against domestic pathogens. “Need for increased efficacy” indicates anticipated increase in therapeutic efficacy with additional antimicrobial use. “Fear of worsening condition” indicates fear of worsening patient condition if only guideline-compliant antimicrobials are used. “Concern about follow-up visits” indicates concern that patients may not return for follow-up despite needing continued monitoring. “Owner pressure for quick recovery” indicates pressure from owner expectations for quick recovery. “Fear of losing clients” indicates fear of losing patients to competing clinics if owners’ demands are unmet. “Difficulty explaining non-prescription” indicates difficulty in explaining reasons for non-prescription of antimicrobials to owners. “Strict adherence to guidelines” indicates adherence to all prescriptions based strictly on guidelines.

Clinical consideration varied among veterinarians that do not strictly adhere to guidelines. Figure 4 indicates that 37.3% of the respondents were primarily concerned about potential health deterioration in patients if they strictly followed prescribing guidelines, whereas 29.3% of the respondents felt pressured to meet owners’ expectations for rapid improvement (Figure 4).

**Figure 5:**
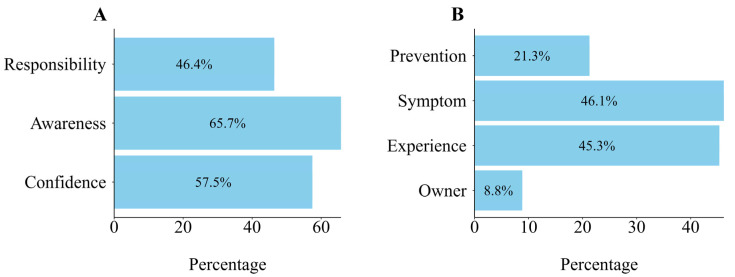
Attitude and practice of veterinarians for prescribing antimicrobials. Note: (**A**) Bar graph illustrating veterinarians’ views on their role in AMR management: I play a key role in preventing antimicrobial resistance (Responsibility); I consider antimicrobial resistance when treating patients (Awareness); I am confident in the evidence supporting my antimicrobial prescriptions (Confidence). (**B**) Reasons for antimicrobial prescription across different clinical/owner scenarios: Prescribing to prevent anticipated infection (Prevention); Prescribing based on observation of clinical signs before confirmation of infection (Symptom); Prescribing based on experience with the condition (Experience); Prescribing in response to owner request (Owner).

An additional survey on veterinarian- and clinical/owner-related factors was conducted to identify the specific predictors of antimicrobial prescription practices and the impact of each predictor (Figure 5). Regarding veterinarian-related factors, most veterinarians acknowledged their significant role in preventing AMR (Figure 5A). The proportion of respondents who are aware of AMR when treating the animal(65.7%) is higher than that of respondents who have confidence in the rationale for prescribing the antimicrobials (57.5%) (Figure 5A). In contrast to previous findings in which owner expectations ranked third, the percentage of veterinarians who stated that they prescribe antimicrobials based on owner demands was low (Figure 5B). Regarding clinical factors, the percentage of veterinarians who prescribe antimicrobials for infection prevention was lower than that of veterinarians who prescribe antimicrobials based on clinical findings prior to the confirmation of infection, or as an empirical practice based on their experience with the disease being treated (Figure 5B).

### 3.7. Measures Implemented to Address Antimicrobial Abuse and Misuse

**Figure 6:**
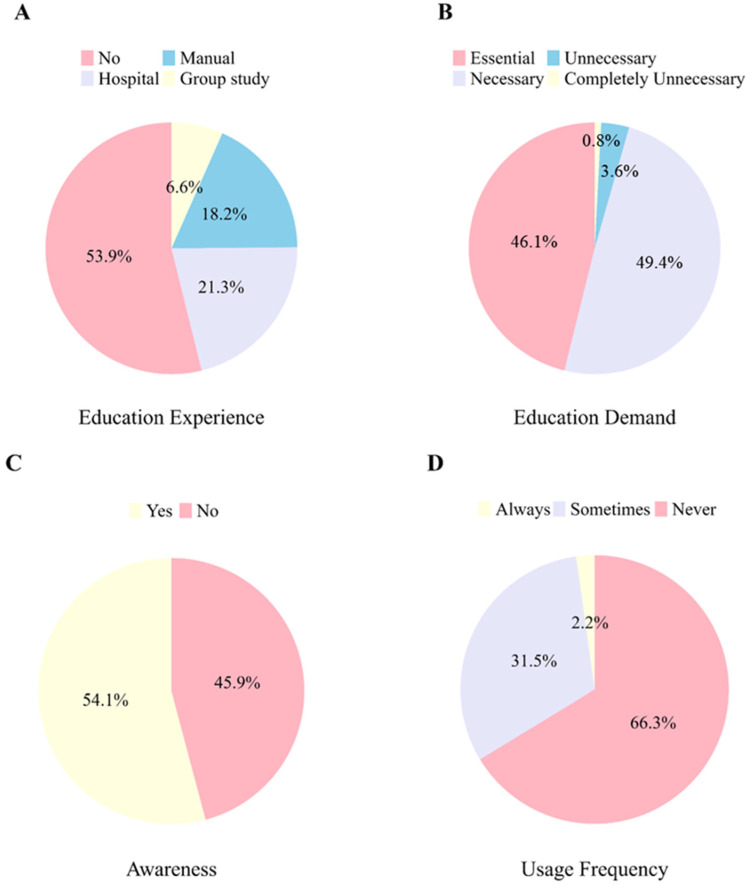
Overview of antimicrobial stewardship education and e-vet system. Note: (**A**) Pie chart for training participation rates and sources of antimicrobial stewardship education. (**B**) Pie chart for responses regarding whether education on antimicrobial stewardship is needed to prevent misuse of antimicrobials. (**C**) Pie chart for responses regarding awareness of the e-Vet system. (**D**) Pie chart for the frequency of e-Vet usage among the participants.

More than half of the veterinarians surveyed (50%) reported not having received any formal training on appropriate antimicrobial use over the past year. Among those who had received training (42.1%), most attended sessions set up by their own veterinary clinics (46.2%), obtained guidance from published protocols (39.5%), and attended group training sessions (14.3%)(Figure 6A).

Most of the respondents (95.5%) agreed that training to prevent antimicrobial abuse and misuse is essential (Figure 6B). Regarding the reasons behind antimicrobial abuse and misuse in companion animals, some respondents cited a lack of awareness of antimicrobial stewardship among veterinarians (32.3%), insufficient guidelines and training (27.9%), and lack of regulatory oversight (13.5%). Approximately 47% indicated that government support should prioritize the development of standardized guidelines, whereas 23.5% called for more training and public awareness initiatives.

Regarding the e-Vet system, 54.1% of the veterinarians acknowledged awareness of the system (Figure 6C); however, only 33.7% reported that they actively use the system (Figure 6D). In addition, 50.8% stated that, although the e-Vet should be utilized to promote antimicrobial stewardship and ultimately reduce AMR, implementing it in clinical practice is challenging. The primary reasons cited were that the e-Vet introduces additional administrative burdens (62.3%) and does not address fundamental issues, such as over-the-counter antimicrobial sales and self-medication (69.8%).

## 4. Discussion

In this study, we determined the species-specific DDDA for active antimicrobial agents using electronic medical record data obtained from 100 veterinary hospitals across Korea (2019–2022). Thereafter, we calculated the DAPD values based on the results and analyzed them according to veterinary clinic size, region, and year. Furthermore, we conducted a survey to assess Korean veterinarians’ awareness of AMR and their antimicrobial prescribing practices. The results indicated a steady increase in antimicrobial usage over time, with higher usage in larger clinics and in clinics in non-metropolitan regions. In addition, the survey revealed that, although veterinarians’ awareness of AMR is generally high, their antimicrobial prescribing decisions are not generally based on strict adherence to guidelines. Clinical factors and pet owner influences were confirmed to impact prescribing practices, likely due to insufficient prescribing guidelines and limited educational support. Although most participants recognized the need for systematic antimicrobial stewardship, opinions regarding the adoption of systems such as e-Vet remained predominantly negative.

The most frequently prescribed antimicrobials included first-generation cephalosporins (28.45%; cefalexin, cefradine, cefadroxil, cefazolin), followed by penicillin (27.13%; amoxicillin-clavulanate, ampicillin), and nitroimidazoles (11.72%; metronidazole). This finding differs slightly from results of previous studies that indicated higher use of cephalosporins, imidazole, penicillin, and tetracyclines [21]. The top three most prescribed antimicrobials in this study align with the primary agents recommended in Korea’s guidelines for the treatment of companion animals [22]. Further, a 2021 Minnesota study on antimicrobial usage revealed that many of the first-line antimicrobials recommended in the guidelines published by the University of Minnesota are frequently prescribed [23,24].

In the present study, the DDDA values for dogs were generally higher than those for cats, with some exceptions for specific antimicrobials. A private Korean survey indicated that dog ownership is more common than cat ownership in Korea, with the average annual veterinary visits for dogs and cats being 5.28 and 4.6, respectively [25]. A study conducted in Australia indicated a higher rate of antimicrobial prescriptions for dogs than for cats, with 145 prescriptions per 1000 dogs compared to 108 per 1000 cats. Another study showed that cat owners tend to be more reluctant to visit a veterinarian than dog owners due to the higher stress sensitivity of cats [26,27]. Considering this finding, the DDDA calculated in the present study is likely to reflect higher healthcare utilization for dogs than for cats; however, further research is needed to confirm this result.

The DAPD calculated from the determined DDDA indicated a generally increasing temporal trend. Considering our study period, the coronavirus disease 2019 (COVID-19) pandemic could partly explain this trend. A previous study showed that demand for veterinary medical care increased during the COVID-19 period due to higher pet adoption rates [28]. In addition, the results indicated that antimicrobial usage in large veterinary hospitals was 1.85 times higher than that recorded in small to medium-sized clinics, and antimicrobial usage in non-metropolitan hospitals was 2.43 times higher than that in metropolitan hospitals. The difference could be attributed to the proportion of patients visiting for preventive medicine. For example, clinics may focus more on providing preventive consultations and medical examinations than hospitals, although specific statistics are lacking in South Korea. Animal owners in urban areas could also have higher motivation than those in rural areas [29]. Interestingly, comparisons performed using gram-based measurements indicated even greater differences, with large hospitals using 3.11 times more antimicrobials than smaller facilities, and non-metropolitan hospitals using antimicrobials 1.51 times more than those in metropolitan areas. These variations depending on the measurement unit used can be attributed to standardization. These findings underscore the utility of DAPD in standardizing measurements categorized according to active agent, allowing for valid comparisons across time periods, hospital sizes, and regions. In fact, standardized metrics are commonly used in human medicine for the comparison of antimicrobial usage across groups according to active agent, institution size, and facility type [30].

The DDDA for companion animals proposed in the present study is a first for Korea; however, it has several limitations. First, the DDDA values are based on data obtained from only 100 conveniently sampled veterinary clinics, out of an estimated 5000 clinics nationwide [31]; thus, the possibility of bias in the results cannot be ruled out. In addition, the study data only captures a partial picture of prescribing practices among veterinarians in Korea, potentially limiting its representativeness. Recent studies have highlighted discrepancies between the WHO’s DDD for humans and actual clinical usage, underscoring the importance of ongoing optimization of standardized metrics to accurately reflect real-world prescribing patterns [32,33]. Therefore, the DDDA values used in this study should be re-evaluated periodically to ensure that they capture actual prescribing practices. Second, the electronic medical records used in this study include only total weights categorized according to species; therefore, the average weights were used to calculate DDDA. Given that the weights of dogs vary widely across breeds, the number of antimicrobials prescribed for each animal varies as well. Using average weights may not fully account for such individual variations, potentially leading to an overestimation of drug exposure in some cases. Thus, the median weight for each species should be used in future calculations of DDDA to improve accuracy. Third, the currently proposed DDDA did not account for critically important antimicrobials for human medicine [34]. Because the AMU of repurposed human medications has a heightened importance from a public health perspective, a separate DDDA measure specifically for these antimicrobials should be developed in future studies.

The results of the survey indicated that, although the veterinarians demonstrated high awareness of AMR, they exhibited comparatively low levels of confidence and accountability regarding antimicrobial prescriptions. In human medicine, targeted clinical pathways and disease-specific treatment guidelines tailored to institutional contexts have been developed and implemented [35]. Additionally, strategies such as decision-support tools that provide real-time feedback and regular audit systems have been proposed to enhance the quality of antimicrobial prescribing [35]. From an educational perspective, clinical practice improvement programs that extend beyond knowledge acquisition, such as mentoring and case-based learning, have been developed to support practical applications [36,37]. Companion animal healthcare could benefit from adopting and adapting similar strategies for the development of tailored interventions that enhance antimicrobial stewardship. Although 50% of practitioners reported that they established their own guidelines, validity and quality control remain uncertain.

The results of this study found that antimicrobial prescription is influenced by factors beyond guidelines, such as clinical factors and pet owner-related factors. A detailed analysis revealed that over 45% of prescriptions were based on veterinarians’ experience and clinical judgment. In other words, veterinarians often prescribe antimicrobials not based on scientific evidence but based on personal experience and beliefs; a similar trend is commonly observed in human medicine [38]. Prescribing antimicrobials empirically and based on symptoms can play a crucial role in timely management of severe infections and improvement of patient outcomes. However, this approach also increases the risk of inappropriate antimicrobial use, as the pathogen is often not accurately identified [39]. To mitigate this issue, accessible antimicrobial susceptibility testing systems should be implemented. Notably, rapid testing methods have been developed in human medicine to address similar concerns [40]. Most of all, regularly updating guidelines to reflect practical scenarios in medicine is imperative for enhancement of prescription accuracy.

Owner-related factors emerged as predictors of antimicrobial prescriptions in this study. In other words, the veterinarians often prescribe antimicrobials in response to owners’ direct or indirect requests. A study conducted in Australia also indicated that approximately 15% of pet owners specifically request antimicrobial prescriptions [41]. In addition, the results of a recent study suggested that pet owners who participate in educational programs to improve awareness understand the impact of AMR better and are more likely to support measures for responsible antimicrobial management [42]. This finding underscores the significant role of pet owners in antimicrobial use, highlighting the need for educational and awareness-raising activities targeted at pet owners as part of AMR prevention efforts.

The findings of this study indicate that veterinarians recognize the importance of monitoring antimicrobial usage but have concerns regarding the increased workload associated with the implementation of the e-Vet system. In human medicine, the introduction of electronic health record systems has facilitated antimicrobial quantification and reduced prescription errors, thus contributing to AMR prevention [35,43,44]. These benefits support the implementation of a similar system in veterinary medicine. Implementing e-Vet systems will enable monitoring and evaluation of appropriate prescribing, increasing data utility and enhancing work efficiency [45]. Thus, from a long-term perspective, implementation of the e-Vet system is essential. Moreover, provision of support through automated reporting systems, decision-support tools, and financial incentives is necessary for alleviating implementation burdens associated with the system. In addition, relevant agencies should actively support veterinarians with regular training programs to ensure effective utilization of the system.

## 5. Conclusions

This study was conducted to increase awareness of antimicrobial use in companion animal healthcare and establish a monitoring framework for antimicrobial usage to address AMR, a significant global health issue. We established a metric for antimicrobial usage in companion animals for the first time in Korea, enabling future comparative evaluations in antimicrobial usage assessments. Additionally, we identified veterinarians’ perceptions of antimicrobial usage and their predictors, providing foundational data for developing educational content and policies to promote antimicrobial stewardship. These multifaceted efforts are expected ultimately to contribute to mitigating the spread of resistant strains within the One Health framework, fostering a sustainable balance among animals, humans, and the environment.

## Figures and Tables

**Table 1:** Veterinary facilities included in this study.

Province	Facility Size		Sum
	Small & Medium	Large	
Seoul	13	30	43
Gyeonggi	12	21	33
Gyeongnam	2	4	6
Jeolla	2	2	4
Gangwon/Gyeongbuk	5	2	7
Chungcheong	4	3	7
**Sum**	**38**	**62**	**100**

**Table 2:** Number of antimicrobial prescriptions, amount of antimicrobials used, and prescription rates in veterinary clinics.

	Total Number of Visits	Visits with Antimicrobial Prescriptions	Antimicrobial Usage (Active Ingredient, g)	Antimicrobial Prescription Rate
**Total (4 years)**	3,110,415	684,153	804,800 g	22.0%
**Annual average**	7776	1710	2012 g	22.0%

**Table 3:** Prescription rates for the most frequently prescribed antimicrobials in Korea (top 15 active agents prescribed).

Atimicrobial Active Ingredient	Prescription Rate	Intended Use
Cefalexin	19.75%	Human, Veterinary
Amoxicillin-Clavulanate	18.08%	Human, Veterinary
Metronidazole	11.72%	Human, Veterinary
Doxycycline	8.43%	Human
Amoxicillin	6.74%	Human, Veterinary
Enrofloxacin	5.51%	Veterinary
Marbofloxacin	4.32%	Veterinary
Cefradine	3.08%	Human
Cefadroxil	3.01%	Human
Cefovecin	2.86%	Veterinary
Cefazolin	2.61%	Human, Veterinary
Ampicillin	2.31%	Human, Veterinary
Clindamycin	1.60%	Human, Veterinary
Trimethoprim-Sulfamethoxazole	1.34%	Human
Cefixime	1.01%	Human

**Table 4:** DDDA categorized according to species and antimicrobial agents prescribed in veterinary clinics in Korea.

Antimicrobial		Species-Specific	
Antimicrobials	Active Ingredient	Canine	Feline
Aminoglycosides			
	Amikacin	173.4	117
	Gentamicin	56.9	38.4
	Neomycin	-	170.2
Amphenicols			
	Chloramphenicol	459.9	274.1
	Florfenicol	252.1	170.2
β-lactam/β-lactamase inhibitors			
	Amoxicillin/clavulanate	212.4	181.4
	Ampicillin/sulbactam	247.4	167
	Piperacillin/Tazobactam	640.3	432.1
	Aztreonam	-	189.3
Carbapenems			
	Meropenem	162	109.4
	Imipenem	126.1	85.1
Cephalosporins			
	Cefazolin	227.3	149.8
	Cefixime	90	68.6
	Cefotaxime	702.7	474.2
	Cefovecin	93.7	63.2
	Ceftiofur	125.7	84.8
	Cefalexin	287.1	193.7
	Cefradine	367.6	271.9
	Cefaclor	131.5	81.6
	Ceftriaxone	315.2	212.7
	Cefadroxil	301.7	191.1
	Cefoxitin	-	170.2
	Ceftazidime	315.2	212.7
	Cefpodoxime	104.6	50
Glycopeptides			
	Vancomycin	169.8	127.6
Imidazoles			
	Metronidazole	164	106.3
Lincosamides			
	Clindamycin	127.9	81.3
	Lincomycin *	126.1	184
Macrolides			
	Azithromycin	106.4	75.2
	Clarithromycin *	63.7	71.5
	Tylosin	216.4	130.8
	Erythromycin	270.8	182.8
	Spiramycin	164.8	106.4
	Tulathromycin	31.5	21.3
Nitrofurantoin			
	Nitrofurantoin	56.9	41.3
Penicillins			
	Amoxicillin	252.4	161.8
	Ampicillin	170.3	98.1
Polymyxin			
	Fosfomycin	504.3	340.3
Quinolones			
	Ciprofloxacin	137.3	104.5
	Enrofloxacin	71.7	47.0
	Levofloxacin	39.8	28.4
	Marbofloxacin	33.0	26.6
	Ofloxacin	115.8	83.5
Sulfonamides			
	Trimethoprim-ulfamethoxazole	227.4	145.6
	Trimethoprim-Sulfamethazine	188.9	127.6
	Trimethoprim-Sulfadiazine	256.1	180.7
Tetracyclines			
	Doxycycline	73.5	51.3
	Minocycline	74.5	59.4
	Tetracycline	330.3	250.7
Nitroimidazole			
	Tinidazole	357.4	214.7
ETC			
	Spiramycin-Metronidazole	77.5	50.4

Note: An asterisk (*) indicates that the DDDA value for cats is higher than that for dogs.

**Table 5:** DAPD categorized according to clinic size and region.

DAPD	2019	2020	2021	2022	Average
**Size**					
Small & Medium	9.2	9.5	11.4	11.6	10.4
large	17	20.4	18.5	20.9	19.2
**Province**					
Seoul	11.5	13.4	15.7	15.3	14
Gyeonggi	10.4	11.7	12.3	13.6	12
Gyeongnam	19	21.8	15.5	15.6	18
Jeolla	9.5	10.3	8.5	11.9	10.1
Gangwon/Gyeongbuk	12.2	14.2	13.3	12.2	13
Chungcheong	16.2	18.2	24.5	29	22
**Average**	**13.1**	**14.9**	**15**	**16.3**	**14.8**

**Table 6:** Demographic characteristics of the survey participants.

	Working Area		
Variable	Seoul	Gyeonggi	Others
**Gender**			
Female	43 (11.9%)	30 (8.3%)	32 (8.8%)
Male	67 (18.5%)	100 (27.6%)	90 (24.9%)
**Age**			
20–29	20 (5.5%)	17 (4.7%)	28 (7.7%)
30–39	61 (16.9%)	64 (17.7%)	49 (13.5%)
40–49	26 (7.2%)	42 (11.6%)	29 (8%)
50–59	2 (0.6%)	5 (1.4%)	15 (4.1%)
more than 60	1 (0.3%)	2 (0.6%)	1 (0.3%)
**Experience**			
less than 1 year	12 (3.3%)	6 (1.7%)	19 (5.2%)
1–5 years	55 (15.2%)	46 (12.7%)	35 (9.7%)
10–20 years	22 (6.1%)	27 (7.5%)	25 (6.9%)
5–10 years	17 (4.7%)	41 (11.3%)	30 (8.3%)
more than 20 years	4 (1.1%)	10 (2.8%)	13 (3.6%)

## Data Availability

The data presented in this study are available by the corresponding author upon reasonable request.

## Supplementary Materials

The following supporting information can be downloaded at: https://www.mdpi.com/article/10.3390/ani15020260/s1, Questionnaire.
