# Supplementary material for: Assessment of Antimicrobial Use for Companion Animals in South Korea: Developing Defined Daily Doses and Investigating Veterinarians’ Perception of AMR
Source: Animals (Basel). 2025 Jan 17;15(2):260. doi: 10.3390/ani15020260 (PMC11758324; doi:10.3390/ani15020260)
Supplement: Supplementary file 1 [file animals-15-00260-s001.zip › animals-3357526-supplementary.pdf]

※ [1-6] Please read the following questions and check the answer that applies to your current status.

1. What is your gender?
   ① Male ② Female
2. What is your age group?
   ① 20s ② 30s ③ 40s ④ 50s ⑤ 60s or older
3. In which region do you work?
   ① Seoul ② Gyeonggi-do (including Incheon) ③ Gyeongsang-do (including Busan, Daegu, Ulsan)
   ④ Jeolla-do (including Gwangju) ⑤ Chungcheong-do (including Daejeon, Sejong)
   ⑥ Gangwon-do ⑦ Jeju-do
4. How many years of experience do you have working in veterinary clinics?
   ① 1 year or less ② 1 - 5 years ③ 5 - 10 years ④ 10 - 20 years ⑤ More than 20 years
5. Please select your area of specialty.
   ① General Practice ② Internal Medicine ③ Surgery ④ Diagnostic Imaging
   ⑤ Emergency and Critical Care
6. What is your experience working in veterinary medical center (5 or more veterinarians)?
   ① None ② Less than 1 year ③ 1-3 years ④ 3-5 years ⑤ 5-10 years ⑥ More than 10 years

※ [7-11] This is a survey on awareness of antibiotic resistance. Please answer to the best of your knowledge.

7. Bacteria resistant to three or more categories of antibiotics are called 'multi-drug resistant bacteria'.
   ① True ② False ③ Not sure
8. Antibiotic-resistant bacteria can be transmitted between companion animals, between companion animals and their owners, and among healthcare workers.
   ① True ② False ③ Not sure
9. I believe that antibiotic resistance in companion animals is a serious public health issue for both humans and animals in our country.
   ① True ② False ③ Not sure
10. If symptoms completely improve during antibiotic treatment, it is okay to stop taking the prescribed antibiotics without completing the full course.
    ① True ② False ③ Not sure
11. Please check all the antibiotics that should be used cautiously in veterinary clinics due to their association with multi-drug resistant bacteria.
    ① Imipenem/Meropenem ② Tetracycline ③ Vancomycin ④ Cefotaxime ⑤ Not sure

※ [12-14] This is a survey on the practice of preventing antibiotic resistance. Please answer to the best of your knowledge.

12. Does your veterinary clinic have a separate isolation facility (ward) for treating patients infected with multi-drug resistant bacteria?
① Yes, we have a separate isolation facility and use it for patients with multi-drug resistant infections and other infectious diseases.
② We have a separate isolation facility, but it is mainly used for patients with infectious diseases (rather than multi-drug resistant infections).
③ We don't have a separate isolation facility, but we treat patients separately depending on the situation.
④ We don't have a separate isolation facility and don't treat patients separately.

13. Antibiotic-resistant bacteria can cause cross-contamination between humans and animals. Do you practice hand disinfection or disinfect equipment before and after treating animals to prevent bacterial infections in your clinic?
① Always ② Most of the time ③ Rarely ④ Almost never

14. Does your veterinary clinic provide training on personal hygiene, facility and equipment disinfection for staff to prevent bacterial infections?
① Yes ② No ③ Not sure

※ [15-26] This is a survey on factors influencing antibiotic prescriptions. Please answer to the best of your knowledge.

15. To what extent do you agree with the following statements when prescribing antibiotics?

- I play an important role in preventing antibiotic resistance: ___
- I consider antibiotic resistance when treating patients: ___
- I am confident in the evidence I use for antibiotic prescriptions: ___
① Less than 20% ② 20-40% ③ 40-60% ④ 60-80% ⑤ More than 80%

16. How often do you prescribe antibiotics in the following situations?

- When infection has not yet occurred but is expected in the future: ____
- When there are clinical signs of infection before confirmation: ____
- Based on treatment experience for the disease: ____
- Due to owner's request: ____
① Less than 20% ② 20-40% ③ 40-60% ④ 60-80% ⑤ More than 80%

17. Does your veterinary clinic have guidelines or protocols for antibiotic prescriptions?
① Yes ② No

18. Please select your first and second most frequently used sources of information when prescribing antibiotics.
(1st choice: ____, 2nd choice: ____)
① Reference materials (textbooks, guidelines, etc.) ② Lectures and guidelines from academic societies/associations
③ Prescription standards of the clinic ④ Personal treatment experience ⑤ Internet ⑥

Opinions of colleagues

19. If you have ever prescribed antibiotics differently from the guidelines you refer to, please select all applicable reasons.
① The antibiotics in the guidelines are not effective against domestic pathogens
② Expectation that additional antibiotics will increase treatment effectiveness
③ Concern that the patient's condition may worsen if prescribed according to guidelines
④ Concern that the patient may not return for follow-up when needed
⑤ Pressure from owners' expectations and demands for rapid improvement
⑥ Concern about losing patients to competing veterinary clinics if unable to meet owners' demands
⑦ Difficulty in explaining to owners why antibiotics are not being prescribed
⑧ I always prescribe antibiotics based on guidelines or protocols

19-2. If you selected any of the reasons ①-⑦ above, what percentage of your total antibiotic prescriptions do these account for?
① Less than 10% ② 10-25% ③ 25-50% ④ 50-80% ⑤ More than 80%

20. Please select all items that you consider to be misuse or overuse of antibiotics.
① Owners giving antibiotics to their pets without a veterinary prescription (self-treatment)
② Prescribing empirical antibiotics without antibiotic susceptibility testing
③ Prescribing prophylactic antibiotics
④ Increasing the dosage when the current antibiotic treatment is ineffective
⑤ Extending the treatment period with the same dosage when the current antibiotic treatment is ineffective
⑥ Prescribing third-line antibiotics (e.g., cefovecin, enrofloxacin, marbofloxacin, vancomycin, carbapenems) as initial treatment
⑦ Discontinuing antibiotic treatment arbitrarily when symptoms improve

21. How often do you use antibiotic susceptibility testing when selecting antibiotics for treating companion animal diseases? (If you select 1 or 2, please proceed to question 22)
① I often request it
② I occasionally request it when treatment is not effective
③ I rarely request it
④ I have never requested it

21-2. If you rarely use antibiotic susceptibility testing, please select the most appropriate reasons (1st choice: ____, 2nd choice: ____)
① I don't know of any reliable testing facilities
② I don't feel the need for testing
③ Owners find the testing costs burdensome
④ I don't feel that prescribing based on test results is effective in treating diseases
⑤ Even if I want to prescribe based on test results, there are often no practical antibiotic options available for animals

22. How often do you use post-operative antibiotics in surgical cases? (If you select 4, please proceed to question 23)
① Always ② Sometimes ③ Rarely ④ Never

22-2. If you use them, please select the most appropriate reason for using post-operative antibiotics.

① As a standard procedure for infection prevention
② Due to frequent experience of post-operative infection complications
③ Only when there is a concern for infection due to extended surgery time, degree of invasiveness, or type of wound
④ Other reasons (________)

23. Have you received any education on reducing unnecessary antibiotic prescriptions or administration in the past year? If so, where did you receive it?
① Did not receive any ② At the veterinary clinic where I work ③ From published guidelines
④ Group education

24. Do you think education is necessary to prevent the misuse and overuse of antibiotics?
① Very necessary ② Necessary ③ Not necessary ④ Not necessary at all

25. What do you think is the biggest factor hindering the proper use of antibiotics in companion animals?
① Lack of regulation and management of antibiotic use
② Lack of awareness among veterinarians about the need for appropriate antibiotic use
③ Lack of awareness among pet owners about the need for appropriate antibiotic use
④ Lack of guidelines and education on antibiotic use
⑤ Other opinions (________)

26. What do you think is the most necessary policy support for appropriate antibiotic use by companion animal veterinarians? (1st choice: ____, 2nd choice: ____)
① Education and promotion of appropriate antibiotic use in companion animals
② Establishment of guidelines for appropriate antibiotic use in companion animals
③ Introduction of an appropriate antibiotic monitoring system
④ Education and promotion of awareness about appropriate antibiotic use for pet owners
⑤ Support for antibiotic susceptibility testing cost
⑥ Operation of reliable microbial testing facilities
⑦ Other opinions (________)

※ [27-30] This is a survey on antibiotic monitoring. Please answer to the best of your knowledge.

[Overview: As antibiotic resistance becomes a serious global issue, countries are implementing antibiotic resistance monitoring systems to prevent antibiotic resistance. In the veterinary field in South Korea, the Veterinary Prescription Management System (e-Vet) is being used to identify inappropriate antibiotic use by veterinarians.]

27. Have you heard of the Veterinary Prescription Management System (e-Vet)?
① Yes ② No

28. Have you ever used the Veterinary Prescription Management System (e-Vet)?
① I always use it
② I use it only when necessary, such as for issuing prescriptions
③ I don't use it

29. Do you think the Veterinary Prescription Management System (e-Vet) is necessary to promote

appropriate antibiotic use and reduce antibiotic resistance?
① It is necessary
② It is necessary but difficult to apply in practice
③ It is unnecessary

29-2. If you think it is unnecessary or difficult to apply, what are the reasons? (Multiple selections possible)
① Unreasonable increase in workload
② Issues with disclosing prescription details
③ Not a fundamental solution (e.g., antibiotic sales through animal pharmacies, self-treatment)
④ Other opinions (________)

30. What policies do you think are necessary to establish the Veterinary Prescription Management System (e-Vet)? (Multiple selections possible)
① Establishing the system through government regulatory policies
② Operating a certification system for veterinary clinics with excellent antibiotic use practices
③ Providing appropriate incentives (e.g., tax exemptions for system use)
④ Reducing administrative procedures (e.g., integration with clinic management software)
⑤ Other opinions (________)

※ Thank you very much for participating in this survey.
